# Spontaneous Formation of Melanin from Dopamine in the Presence of Iron

**DOI:** 10.3390/antiox9121285

**Published:** 2020-12-16

**Authors:** David M. Hedges, Jordan T. Yorgason, Andrew W. Perez, Nathan D. Schilaty, Benjamin M. Williams, Richard K. Watt, Scott C. Steffensen

**Affiliations:** 1 Enterprise Information Management, Billings Clinic, 2800 10th Avenue North, Billings, MT 59101, USA; dhedges@billingsclinic.org; 2 Department of Chemistry and Biochemistry, C100 BNSN, Brigham Young University, Provo, UT 84602, USA; rwatt@chem.byu.edu; 3 Department of Physiology and Developmental Biology, 4005 LSB, Brigham Young University, Provo, UT 84602, USA; jordanyorg@byu.edu; 4 Neuroscience Program, S-192 ESC, Brigham Young University, Provo, UT 84602, USA; awperez@mcw.edu (A.W.P.); schilaty.nathan@mayo.edu (N.D.S.); bmw76@byu.edu (B.M.W.); 5 Department of Psychology, 1001 KMBL, Brigham Young University, Provo, UT 84602, USA; 6 Department of Orthopedic Surgery, Mayo Clinic, 200 First Street SW, Rochester, MN 55905, USA; 7 Department of Physiology & Biomedical Engineering, Mayo Clinic, 200 First Street SW, Rochester, MN 55905, USA

**Keywords:** dopamine, melanin, neuromelanin, oxidative stress, iron

## Abstract

Parkinson’s disease is associated with degeneration of neuromelanin (NM)-containing substantia nigra dopamine (DA) neurons and subsequent decreases in striatal DA transmission. Dopamine spontaneously forms a melanin through a process called melanogenesis. The present study examines conditions that promote/prevent DA melanogenesis. The kinetics, intermediates, and products of DA conversion to melanin in vitro, and DA melanogenesis under varying levels of Fe^3+^, pro-oxidants, and antioxidants were examined. The rate of melanogenesis for DA was substantially greater than related catecholamines norepinephrine and epinephrine and their precursor amino acids tyrosine and l-Dopa as measured by UV-IR spectrophotometry. Dopamine melanogenesis was concentration dependent on the pro-oxidant species and Fe^3+^. Melanogenesis was enhanced by the pro-oxidant hydrogen peroxide (EC_50_ = 500 μM) and decreased by the antioxidants ascorbate (IC_50_ = 10 μM) and glutathione (GSH; IC_50_ = 5 μM). Spectrophotometric results were corroborated by tuning a fast-scan cyclic voltammetry system to monitor DA melanogenesis. Evoked DA release in striatal brain slices resulted in NM formation that was prevented by GSH. These findings suggest that DA melanogenesis occurs spontaneously under physiologically-relevant conditions of oxidative stress and that NM may act as a marker of past exposure to oxidative stress.

## 1. Introduction

Melanin is a broad term for a group of natural pigments formed by the oxidation of amino acids or catecholamines followed by polymerization into an insoluble pigment. Melanocytes (cells expressing melanin pigments) are found in the skin [1], the eye (in both the iris pigment epithelium and in the retinal pigment epithelium) [2], the inner ear [3,4], in the olfactory mucosa [5], and in dopamine (DA) cells in the substantia nigra [6]. Genetic disorders of melanogenesis (including albinism) can be characterized by a lack of functional tyrosinase, the enzyme that produces melanin. This results in lower levels of melanin in the skin, eye, ear, and nose epithelium, but not in the brain, indicating that neuromelanin (NM) in the brain forms through an alternative mechanism. Besides melanin’s photoprotective properties, it is known to act either as a pro-oxidant or an antioxidant depending upon its degree of polymerization [7].

Neuromelanin is a brown to black melanin found in specific populations of catecholaminergic neurons in the brain, including noradrenergic neurons in the locus coeruleus (LC) and dopaminergic (DAergic) neurons in the substantia nigra pars compacta (SNc) in the midbrain of humans and some non-human primates [6,8]. This is what gives the SNc its distinctive dark color. Parkinson’s disease (PD), the second most common neurodegenerative disease, is characterized by motor deficits such as muscle rigidity, muscle tremors, slow planned movements, and postural instability [9]. The primary cause of these motor deficits is due to the loss of DA neurons in the nigrostriatal pathway, which consists of DA neurons of the SNc projecting to target regions of the striatum [10]. Due to the correlation between the death of the pigmented DA cells in the SNc and PD symptoms, it was originally thought that NM was toxic and resulted in the death of DA cells in PD. However, the relationship of NM in SNc DA neurons to PD pathogenesis is much more complicated: DA neurons containing less NM are more likely to die in models of PD [11,12] and NM can bind to and render toxic iron ions redox inactive [13]. Given the emerging evidence that oxidative stress plays a large role in PD pathogenesis [14,15,16], NM may in fact have a protective role. However, this means that NM holds a repository of redox inactive iron and potentially other heavy metals; if spilled into the extracellular space (for example, due to apoptosis), NM can degrade resulting in a sudden release of toxic metals and inflammatory response [17]. Thus, while NM may have a neuroprotective role [18], it can also lead to a cascade of DA cell death once one cell dies and releases its NM reservoirs [17].

In the skin, melanin is formed enzymatically by tyrosinase [6], but mutations in tyrosinase that prevent melanin formation in the skin do not appear to affect the formation of NM. Importantly, NM deposits in the SNc are not ubiquitous across age groups in humans [19], and generally are only present after infancy and childhood. Since melanogenesis of NM does not appear to be linked to enzymes already associated with melanogenesis, the formation of NM may occur via non-enzymatic processes such as aggregation through coordination chemistry or radical polymerization [20]. In this study, the spontaneous melanogenesis of DA was investigated to determine the potential role of direct and indirect Fe^3+^ interactions with DA and radical polymerization. We hypothesized that spontaneous melanogenesis of DA would be dependent on the presence of Fe^3+^, either through DA aggregation through the catechol’s coordination chemistry with Fe^3+^ or through radical intermediates generated via the Fenton reaction, and that the anti-oxidants ascorbate and glutathione would block melanogenesis.

## 2. Materials and Methods

### 2.1. Reagents and General Procedures

To prevent premature melanogenesis, DA solutions were prepared immediately before each experiment. Solutions used were physiologically-relevant and included intracellular fluid (ICF) consisting of: 125.0 mM KCl, 10.0 mM HEPES, 2.0 mM MgCl_2_, and 0.5 mM CaCl_2_ and extracellular fluid (ECF) consisting of: 124.0 mM NaCl, 2.0 mM KCl, 1.25 mM NaH_2_PO_4_, 24.0 mM Na_2_HCO_3_, 12.0 mM Glucose, 1.2 mM MgSO_4_, and 2.0 mM CaCl_2_). Both ICF and ECF were bubbled with 95% O_2_/5% CO_2_ to help maintain pH 7.3–7.4. In some experiments, Ca^2+^ was either not added or added at different concentrations, as discussed in the Results section. The concentration of DA was 30 µM in all experiments, except when otherwise specified. Although ECF and ICF were used as buffers to mirror extracellular and intracellular conditions, the focus of this study was to monitor the chemical reaction of DA conversion to melanin within physiologic parameters, thus the role of metal ions in DA melanogenesis was evaluated at concentrations across their known cellular ranges.

All reagents were purchased from the suppliers and were used at the concentrations indicated in the Results: 3-hydroxytyramine (DA; Sigma-Aldrich, St. Louis, MO, USA), sodium ascorbate (Spectrum Chemical, New Brunswick, NJ, USA); glutathione (GSH; Sigma-Aldrich, St. Louis, MO, USA); hydrogen peroxide (Fisher Scientific, Pittsburgh, PA, USA); desferrioxamine (Sigma-Aldrich, St. Louis, MO, USA); transferrin (Lee Biosolutions, Maryland Heights, MO, USA).

### 2.2. Preparation of Dopamine Solutions

To ensure that there was no iron contamination in the solutions, molecular biology grade water was treated with 10 µM CO_3_^2−^ and 10 µM transferrin. Carbonate facilitates iron loading onto transferrin, which is a protein that binds Fe^3+^ higher than 10^20^ M^−1^ and has a molecular weight of 80 kDa [21,22]. After a 15 min incubation, the solutions were loaded into Amicon Ultra-4 Centrifugal Filter Units with Ultracel-10 membrane, with a molecular cut-off at 10 kDa (EMD Millipore) and spun in a centrifuge at 4000 rpm for 15 min. The transferrin was retained in the upper chamber of the filter and the filtrate (purified water) was recovered from the flow-through of the filter and used for experiments. Transferrin-treated water was used in all solutions except for determining the role of the initial concentration of DA and the effect of Fe^3+^ and desferroxamine (DES) on the rate of melanogenesis.

### 2.3. UV-Vis Spectrophotometry and the 480 nm Assay

Samples were placed into standard 10 mm light path quartz cuvettes (US Solid, Oakland, CA, USA) and the full spectrum was scanned (200–900 nm) in a Thermo Scientific Biomate 3S UV-Visible Spectrophotometer (Waltham, MA, USA). For experiments that were required to be iron-free, two-sided plastic disposable cuvettes (VWR) were used in place of quartz. An initial scan of the sample was taken immediately after sample preparation and collections continued every 10 min for two hours (inclusive, 13 collections total). These experiments, and those of others [23,24], have demonstrated that the process of melanogenesis of catecholamines can be closely monitored in its early stages by the formation of an absorbance peak at 480 nm.

### 2.4. Animal Subjects

Male C57BL/6 mice (PND 60-90) were bred and cared for in accordance with the National Institutes of Health Guide for the Care and Use of Laboratory Animals. At weaning (PND 21), animals were housed on a reverse light/dark cycle (lights on from 8 p.m. to 8 a.m.) in groups of 2–5/cage and given ad libitum access to food and water. Experimental protocols were approved by the Brigham Young University Institutional Animal Care and Use Committee (IACUC # 18-1202, approved 10/29/2020).

### 2.5. Brain Slice Preparation

Mice were anesthetized with isoflurane (5%), decapitated, and brains were rapidly dissected and sectioned into 400 µm slices into artificial cerebrospinal fluid (ACSF) consisting of (in mM): 124 NaCl, 2 KCl, 1.25 NaH_2_PO_4_, 24 NaHCO_3_, 12 Glucose, 1.2 MgSO_4_, 2 CaCl_2_, pH 7.3, which was bubbled with 95% O_2_/5% CO_2_ and maintained at 34–36 °C. Slices were then transferred to a recording chamber with continuous ACSF flow (2.0 mL/min) maintained at 34–36 °C. The NAc was visualized at the level of the anterior commissure under low magnification with Nikon Diaphot inverted microscopes in the transmitted light mode and Olympus X51 microscopes with transmitted infrared Dodt gradient contrast imaging.

### 2.6. Carbon Fiber Electrodes, Calibration, and Fast Scan Cyclic Voltammetry

For fast scan cyclic voltammetry (FSCV) recordings, carbon fiber electrodes were prepared as previously described [25]. Briefly, a 7.0 µm diameter carbon fiber was inserted into borosilicate glass capillary tubing (1.2 mm outer diameter; A-M Systems, Sequim, WA, USA) under negative pressure and subsequently pulled on a vertical pipette puller (Narishige, East Meadow, NY, USA). The carbon fiber electrode (CFE) was then cut under microscopic control with 150 µm of bare fiber protruding from the end of the glass micropipette. The CFE was back-filled with 3 M KCl. The CFE’s were regularly calibrated with known concentrations of DA. With the CFE positioned in the solution in a closed system of extracellular fluid (ECF) bubbled with 95% O_2_/5% CO_2_, a known concentration of DA was superfused at a high flow rate (5 mL/min) past the electrode and the maximum nA signal produced by DA was observed. These DA calibrations were averaged to convert a nA signal of DA oxidation to µM concentration of DA.

To observe melanogenesis of DA utilizing FSCV, 3.0 mL of ECF was placed in a chamber (Warner Instruments, Hamden, CT, USA) that was modified to prevent leakage (i.e., static flow system) and bubbled with 95% O_2_/5% CO_2_ gas to maintain pH at 7.2–7.4. Both a reference electrode and the CFE were positioned well below the surface of the fluid, but off the bottom of the chamber for in vitro recordings of DA melanogenesis and 100–200 μm below the surface of the tissue slice for ex vivo recordings. The electrode potential was linearly scanned as a triangular waveform from −0.4 to 1.2 V vs. Ag/AgCl using a scan rate of 400 V/s and a 1 Hz collection frequency for 1.5–2 h. Cyclic voltammograms were recorded at the CFE every 1 s by means of a ChemClamp voltage clamp amplifier (Dagan Corporation, Minneapolis, MN, USA). Voltammetric recordings were performed and analyzed using LabVIEW (National Instruments, Austin, TX, USA)-based customized software (Demon Voltammetry [26]). The recording was initiated without DA being present in solution (to allow for an appropriate baseline recording—FSCV is a background-subtracted technique). Dopamine was added to the bath chamber from a stock solution (10 mM in 0.1 N perchloric acid) after 5.0 min of baseline recording at a volume to produce 30 µM in 3.0 mL of ECF for in vitro recordings and the tissue was stimulated first with local electrical stimulation to assure a DA signal and then by depolarization with 100 mM KCl.

### 2.7. NMR Studies

For NMR experiments, 30 μM DA was pipetted into standard Norrell NMR tubes (Landisville, NJ, USA). The samples were analyzed using a Varian NMR-System 500 MHz (Agilent Technologies, Santa Clara, CA, USA). Solutions were prepared in D_2_O (Cambridge Isotope Laboratories, Andover, MA, USA) to minimize solvent noise.

### 2.8. Statistical Analyses

The results were compiled from spectrophotometers and the rate of melanogenesis was calculated from the slope of the absorbance versus time. For FCSV experiments, peak DA oxidation currents measured via FSCV were isolated and compiled. The means were then grand-averaged across trials. Values were expressed as means ±SEM for cumulated data. Between-subject group comparisons were analyzed via one-way ANOVAs. The criterion of significance was set at *p* < 0.05 (*), *p* < 0.01 (**), *p* < 0.001 (***), and *p <* 0.0001 (#). All statistics were calculated with IBM SPSS Statistics 21 (Armonk, New York, NY, USA).

## 3. Results

### 3.1. Role of Initial Substrate in Rate of Melanin Formation

**Figure 1:**
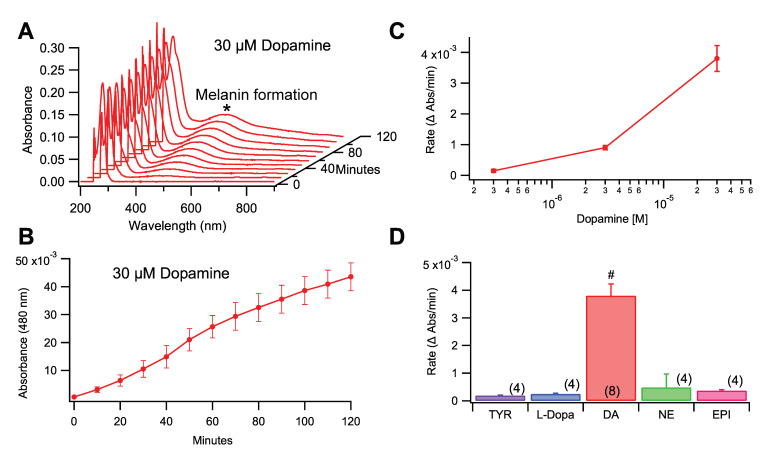
Spectrophotometric characterization of DA melanogenesis, sensitivity to initial substrate concentration, and comparison with DA precursors and metabolites. (**A**) Waterfall plot of full spectrum (190–900 nm) absorbance demonstrating the progressive melanogenesis of DA at 30 μM. The 480 nm peak indicated with an * represents DA melanogenesis. (**B**) This graph summarizes the kinetics of the 480 nm peak (*n* = 8) over two hours representing an increase in the DA melanogenesis. (**C**) The rate of growth (Δ Abs/min) of DA melanogenesis (given by the growth of an absorbance peak at 480 nm over a two-hour collection interval as determined by linear regression) was dependent on initial DA concentration. (**D**) This graph compares the rate of growth of DA melanogenesis to that produced by its amino acid precursors and metabolic derivatives (all at 30 µM concentration): tyrosine (Tyr); l-dopa; norepinepherine (NE); and epinephrine (EPI). While all species tested show melanogenesis, DA polymerizes to melanin at a significantly faster rate. Values in parentheses represent *n* values. Hashtag # indicates significance level *p* < 0.0001. Data is presented as mean ±SEM.

Dopamine in aqueous solution converts to melanin spontaneously over time and can be tracked by measuring the change in absorbance at 480 nm (Figure 1A,B). To determine if the formation of melanin was dependent on the initial concentration of DA, the rate of melanogenesis was examined at 0.3, 3.0, and 30 μM DA (Figure 1C). These concentrations are significantly lower than vesicle concentrations of 190–300 mM [27,28], but are comparable to intracellular and synaptic concentrations. The results demonstrated that the rate of melanin formation was directly proportional to the initial concentration of DA in solution, with higher concentrations of DA resulting in faster rates of melanin formation. This relationship had a strong linear correlation (R^2^ = 0.987). As the 30 μM concentration gave a faster and obvious change that could be easily monitored in a reasonable timeframe, this concentration was utilized in further experimentation. The rate of melanogenesis (rate of growth of the 480 nm absorbance peak) produced by DA to that of melanins produced by tyrosine, l-DOPA, epinephrine, and norepinephrine was compared at equimolar concentrations (30 µM). Dopamine showed the fastest rate of melanogenesis when compared with the related compounds (Figure 1D; one-way ANOVA: *F*_(4,19)_ = 30.734, *p* < 0.0001). Tukey post-hoc analysis did not demonstrate any significance between any groups aside from DA (*p* > 0.05).

To verify that the 480 nm peak measured with spectrophotometry was melanin or a melanin precursor (such as 5, 6-dihydroxyindole or dopaminochrome), we performed proton nuclear magnetic resonance (H-NMR) experiments of the DA solution in ECF buffer (to minimize solvent noise, D_2_O was used instead of H_2_O). Using H-NMR with Fe^3+^ (10 µM) present to maximize melanogenesis, a kinetic study was performed. A comparison of the NMR peaks showed that the predominant species in solution at both time zero and 2 h was DA (melanin is inherently insoluble and precipitates out of solution upon formation) (Supplemental Figure S1). This indicated that the colorimetric change tracked with the 480 nm assay was either melanin or an immediate precursor to melanin.

### 3.2. Role of Fe^3+^ in Rate of Dopamine Melanogenesis

**Figure 2:**
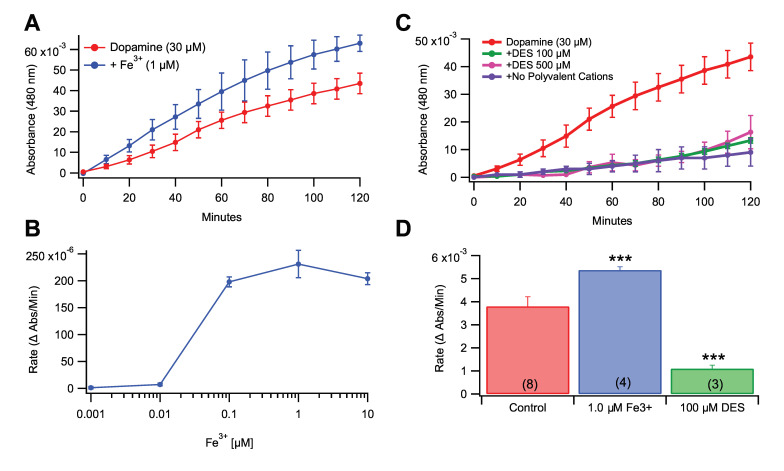
Effects of Fe^3+^ levels on DA melanogenesis. (**A**) Fe^3+^ increased the DA melanogenesis in a concentration-dependent manner in transferrin-treated, iron-free water, as untreated ECF has a trace of Fe^3+^. (**B**) Dose-response curve for growth rates of DA melanogenesis based on Fe^3+^ concentration. The curve is roughly sigmoidal with a precipitous rise between 0.01 and 0.1 μM and with an EC_50_ of ~0.03 μM Fe^3+^. (**C**) Comparison of DA melanogenesis rates of 1.0 µM Fe^3+^ and 30 μM DA (Control) vs. 100 µM and 500 µM DES (iron chelator) vs. no polyvalent cations. (**D**) Fe^3+^ significantly increased the rate of melanogenesis while DES decreased it. Values in parentheses represent *n* values. Asterisks *** indicate significance level *p* < 0.001. Data is presented as mean ±SEM.

It has been proposed previously that NM may act as an iron chelation agent in DA neurons and NM contains two iron chelation sites [29,30]. Thus, evaluation was performed of the rate of DA melanogenesis in the presence of FeCl_3_ at five concentrations (1.0, 10.0, 0.1, 1.0, and 10.0 μM; Figure 2) mixed with the reaction mixture (transferrin-treated ddH_2_O + 30 µM DA). Iron significantly increased the rate of DA melanogenesis in a concentration-dependent manner (Figure 2A,B), whereas 100 and 500 µM DES, an iron-specific chelating agent, decreased the rate of melanogenesis (Figure 2C; *F*_(5,20)_ = 90.812, *p* < 0.0001). All conditions compared against the DA control by one-way ANOVA showed significance between DA vs. iron (1.0 µM) as well as DA vs. DES (100 µM; Figure 2D; *F*_2,12_ = 27.12, *p* < 0.0001).

### 3.3. Role of Ca^2+^ in Rate of Dopamine Melanogenesis

Like Fe^3+^, Ca^2+^ is known to form coordinate bonds with the hydroxyl groups of catechols [31] and is present in chemically-relevant amounts in the striatum. Thus, six concentrations of Ca^2+^ were tested (0.1, 1.0, 10.0, 100, 1.0, and 10.0 mM) in transferrin-treated solutions to determine Ca^2+^ induction of melanogenesis. Calcium did not significantly induce melanogenesis, and was significantly reduced compared to the Fe^3+^ exposed controls (Supplementary Figure S2; *F*_(6,19)_ = 25.63, *p* < 0.0001).

### 3.4. Effects of Pro-Oxidants and Antioxidants in Rate of Dopamine Melanogenesis

**Figure 3:**
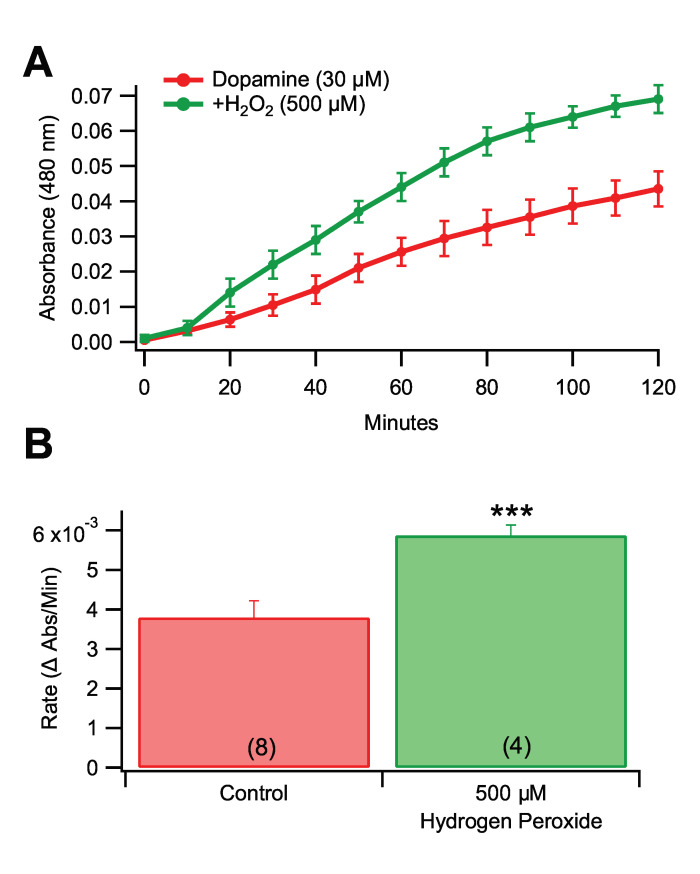
The pro-oxidant H_2_O_2_ promotes melanogenesis of DA. (**A**) Hydrogen peroxide (500 µM) increased DA melanogenesis compared to 30 µM DA alone. (**B**) Hydrogen peroxide significantly increased the rate of DA melanogenesis. Values in parentheses represent *n* values. Asterisks *** indicate significance level *p* < 0.001. Data is presented as mean ±SEM.

**Figure 4:**
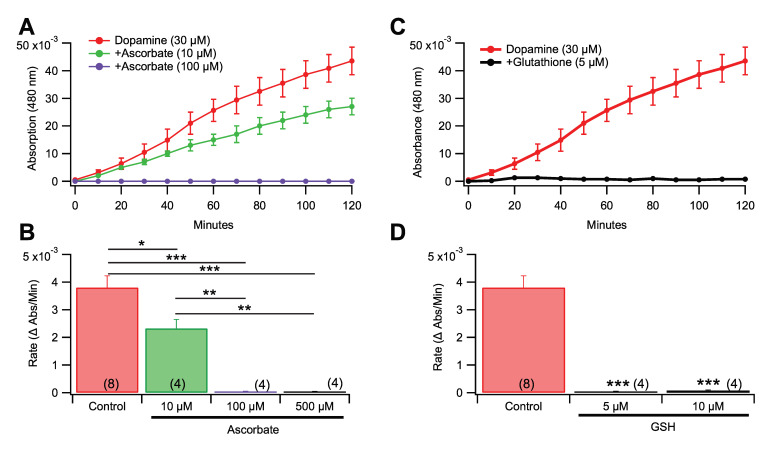
Effects of anti-oxidants on melanogenesis of DA. (**A**) Concentration response demonstrating that increased ascorbic acid (ASC) concentrations reduce and block DA melanogenesis. (**B**) Ascorbate significantly reduced the rate of DA melanogenesis. (**C**) Glutathione markedly reduced DA melanogenesis. Note that these concentrations are three orders of magnitude lower than those of ASC, and much lower than GSH levels normally seen in cells. (**D**) Glutathione significantly reduced the rate of DA melanogenesis. Values in parentheses represent *n* values. Asterisks *, **, *** indicate significance level *p* < 0.05, *p* < 0.01 and *p* < 0.001 respectively. Data is presented as mean ±SEM.

To confirm that oxidative agents can drive the conversion of DA to melanin, we added H_2_O_2_ (500 µM) into the reaction mixture. This concentration of H_2_O_2_ increased the rate of melanogenesis by 54.6% (Figure 3A,B; *F*_(1,10)_ = 27.422, *p* = 0.0004). To provide a correlate for the experiment with the pro-oxidant H_2_O_2_, the effects of the antioxidants ascorbate (ASC) and glutathione (GSH) on the rate of DA melanogenesis were tested. Ascorbate suppressed the formation of melanin at 10 µM or higher concentrations (Figure 4A,B; *F*_(3,16)_ = 36.009, *p* < 0.0001), with clear concentration dependent effects and an approximate IC_50_ of 10 µM. Individual dose comparisons demonstrated that 10 µM ASC, 100 µM ASC, and 500 µM ASC were each significant when compared against the non-antioxidant control (Dunnett’s multiple comparison; respectively, *q* = 7.406, *p* < 0.001; *q* = 7.886, *p* < 0.001; *q* = 7.921, *p* < 0.001). Glutathione had a similar, but more efficient, suppressing action on DA melanogenesis at concentrations (Figure 4C,D; One way ANOVA; F_(2,13)_ = 37.89, *p* < 0.0001) as low as 5 and 10 μM (Dunnett’s multiple comparison; 5 μM: *q* = 7.116, *p* < 0.001); 10 μM: *q* = 7.099, *p* < 0.001), and ineffective at lower concentrations (1–3 μM) from a separate set of experiments (Supplementary Figure S3; *F*_4,19_ = 12.69, *p* < 0.0001). Neuromelanin is found inside DA neurons. Since the previous experiments were run in ECF buffer, we validated the effects of 10 µM Fe^3+^ and 5 µM GSH by replicating them using an ICF buffer. Melanogenesis was observed in ICF buffer (*F*_(2,9)_ = 228.715, *p <* 0.0001), thus melanogenesis happens under ICF and ECF conditions.

### 3.5. Fast-Scan Cyclic Voltammetry of Dopamine Melanogenesis

**Figure 5:**
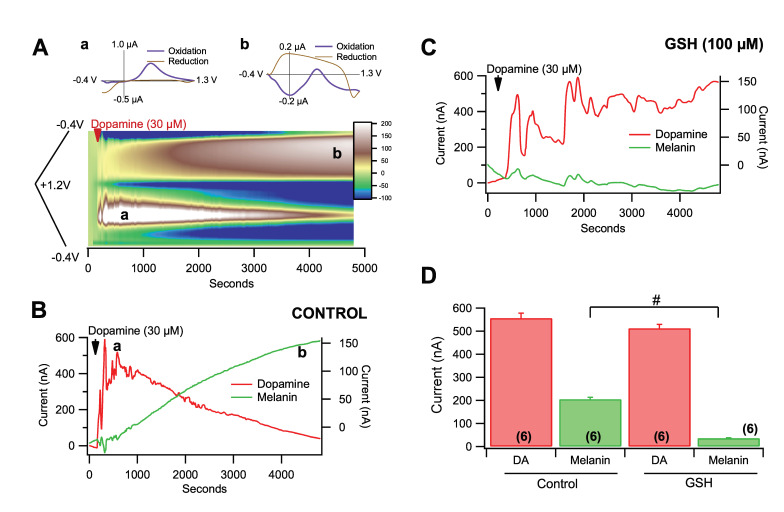
Fast-scan cyclic voltammetry of melanogenesis of DA in vitro. (**A**) Insets show representative cyclic voltammograms at the points indicated on the graph during equilibration (Aa) of DA (30 µM) and following partial conversion of DA to melanin (Ab) in a closed system (3 mL of ECF). The characteristic oxidation peak at 0.5 V and reduction peak at −0.38 V are evidence of DA (Aa). The DA redox peaks are still visible (0.5 V and −0.38 V) more than 2 h after equilibration (Ab), but significantly reduced, indicating that DA is still in solution, but the redox signal slowly decays as the signal from melanin grows (a broad peak extending from −0.02 V and a sharper peak at −0.4 V). The colorplot below shows currents on the color *z*-axis for cyclic voltammograms over time at 1 Hz sampling rate. The voltammograms in Aa and Ab were taken at the times a,b indicated on the plot, demonstrating the peak and fall of DA and the progressive emergence of melanin. (**B**) This graph shows the current over time for DA vs. melanin in the same example shown in A. (**C**) In this representative example, 100 μM GSH not only blocked the formation of melanin by 30 µM DA but the decay in DA over time. (**D**) Cumulative comparison of spontaneous melanogenesis as measured by FSCV. GSH significantly reduced DA melanogenesis. Significance marker # denotes *p* < 0.0001. Data is presented as mean ± SEM.

**Figure 6:**
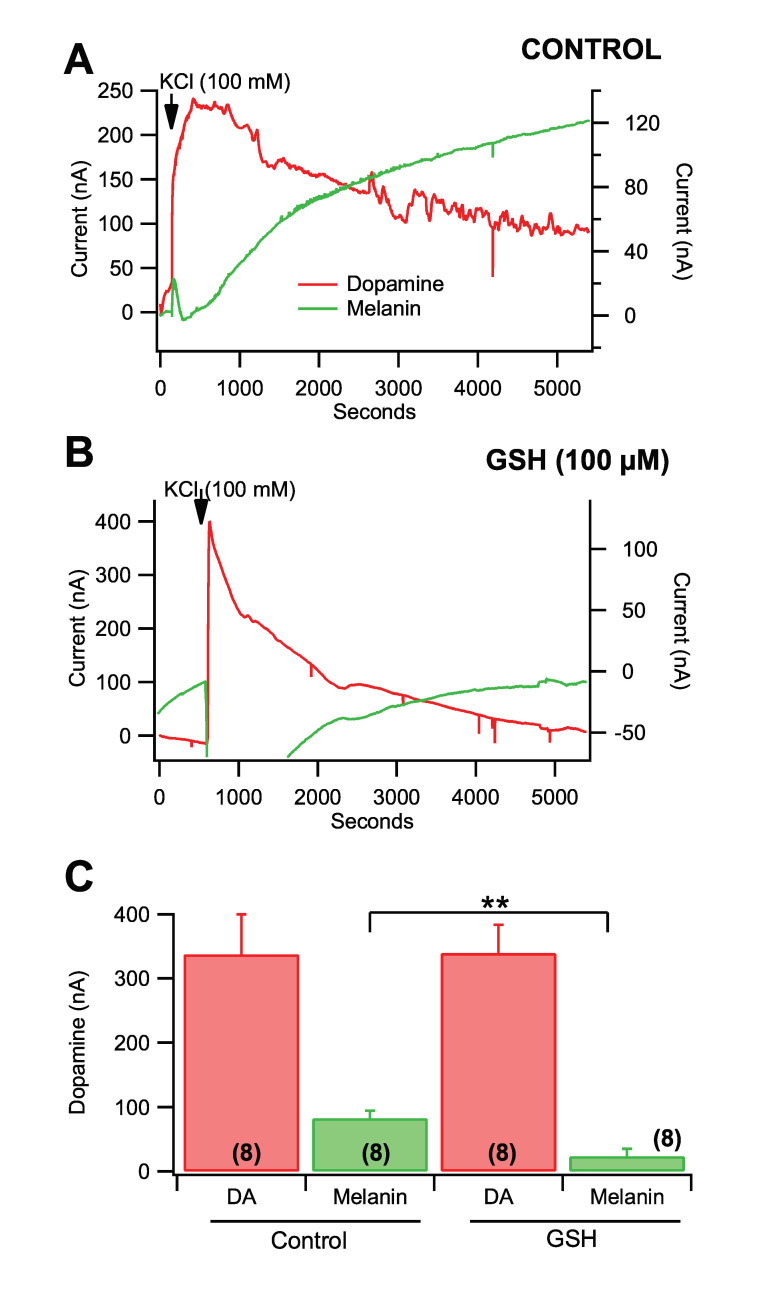
Fast-scan cyclic voltammetry of melanogenesis of DA in the NAc slice preparation ex vivo. (**A**) This graph shows the current over time for DA vs. melanin after depolarization of DA terminals in the NAc produced by infusion of 100 mM KCl into the closed system. (**B**) In this representative example, 100 μM GSH reduced the formation of melanin by KCl but not the decay in DA over time, likely due to ongoing re-uptake of DA at DA terminals in the striatum. (**C**) Cumulative comparison of spontaneous melanogenesis as measured by FSCV. GSH significantly reduced DA melanogenesis. Significance marker ** denotes *p* < 0.01. Data is presented as mean ±SEM.

To confirm the spectrophotometric recordings of the conversion of DA to melanin and the role of select antioxidants, replication of previous findings was performed using FSCV. The voltage ramp was sufficient to measure both the concentration of DA and the concentration of melanin simultaneously (Figure 5A). Two conditions were run to confirm the previous spectrophotometry findings. First, DA was added into 3 mL of ECF buffer for a resulting concentration of 30 µM and monitored for the conversion of DA to melanin over 1–2 h (Figure 5A). The DA signal reached a peak in seconds and slowly decayed over time concomitantly with an increase in melanin formation (Figure 5B). When GSH (100 µM) was combined with 30 µM DA in ECF buffer, the DA signal did not decrease over time and melanin formation was markedly reduced (Figure 5C). Glutathione significantly reduced melanogenesis (Figure 5D; *F*_(1,11)_ = 117.7, *p* = 7.5 × 10^−7^) without having a significant effect on peak DA levels (*p* > 0.05). To confirm that melanin can form from DA in biological systems as opposed to synthetic DA in vitro, we evaluated the effects of GSH in a static system ex vivo containing ACSF. Five minutes into the collection, we added 100 mM KCl to evoke massive DA release from DA terminals by depolarization. This level of KCl evoked DA release at concentrations ranging from 10–30 µM ex vivo (Figure 6A; red). Just as we observed with chemically manufactured DA, we observed a DA peak arise early and decay as a melanin peak rose later in the collection (Figure 6A; green). When GSH (100 µM) was combined with 30 µM DA, the KCl-evoked DA signal peaked and decreased over time (Figure 6B; red) and melanin formation was markedly reduced (Figure 6B; green). Unlike in vitro, GSH did not prevent DA decrease over time likely due to uptake in the slice by the DA transporter. Glutathione significantly reduced melanogenesis (Figure 6C; *F*_(1,15)_ = 15.1, *p* = 0.002) without having a significant effect on peak DA levels (*p* > 0.05).

## 4. Discussion

Dopamine spontaneously polymerizes into melanin, with the rate of formation having a positive linear correlation with initial DA concentration. This corresponds with the in vivo experiments done by Sulzer et al., indicating that the intracellular synthesis of NM is dependent on the cytosolic concentration of DA (DA not sequestered in vesicles) [32]. Dopamine and chemically similar molecules such as DA precursors tyrosine and l-Dopa, as well as the catecholamines norepinephrine and epinephrine also undergo spontaneous melanogenesis, but at a much slower rate than DA. Important chemical characteristics appear to be the combined presence of a catechol and a primary amine located on an alkane chain of at least two carbons (corresponding with a high degree of molecular flexibility).

Using UV-visible spectrophotometry, we initially developed a spectrophotometric assay that measures the progressive formation of melanin by quantifying the rate of polymerization based on the absorbance over time at 480 nm. Next, the optimal chemical environment for spontaneous melanogenesis of DA was examined, focusing on the possible role of the catechol functional group of DA-coordinating cations, specifically free electrons and Fe^3+^ and Ca^2+^ as both these ions are easily coordinated by catechol groups [33]. Pro-oxidants Fe^3+^ and H_2_O_2_ increased the rate of melanogenesis, whereas Ca^2+^ had no effect on melanogenesis. Furthermore, antioxidant species such as ascorbate and GSH blocked melanogenesis, indicating that there is a strong radical component to melanogenesis and that coordination chemistry alone (through the redox-inactive Ca^2+^) is insufficient to drive melanogenesis, and this interaction is specific to DA over other catecholamines and tyrosine.

In DA neurons, NM deposits have a significant amount of Fe^3+^ bound in a non-reactive state [13,34]. Based on this observation, it was estimated that Fe^3+^ may speed up the rate of melanogenesis, but not as a catalyst, as free Fe^3+^ would be consumed in the reaction. The results indicated that Fe^3+^ ions significantly increased the rate of melanogenesis (EC_50_ = 50 μM), and that chelation of free Fe^3+^ by DES correspondingly decreased melanogenesis. Interestingly, not only can Fe^3+^ coordinate the catechol of DA, but it can also generate free electron radicals via the Fenton reaction, lipid peroxidation, and by catalyzing superoxide formation [35,36]. This indicates that Fe^3+^ may facilitate melanogenesis by simply aggregating DA molecules (the coordination structure of Fe^3+^ affords near perfect geometry for coordinating three catechols [37,38]), initiating a radical polymerization cascade, or potentially a combination of coordination and radical chemistry. Dopamine metabolism generates hydroxyl radicals and DOPAL when degraded by monoamine oxidase A [39,40], and the SNc contains higher levels of iron as compared to other brain areas [35]. These iron deposits are found in the midbrain as well as in DA cell culture [41], indicating that iron is localized in DA somata and not just in glia. Iron is a significant source of oxidative stress, particularly when combined with H_2_O_2_ produced from DA metabolism, creating hydroxyl radicals via the Fenton reaction [42]. The natural metabolism of DA by monoamine oxidase generates H_2_O_2_ [43], highlighting the potential importance of NM acting as an antioxidant. Neuromelanin possesses a stable radical, enabling direct radical-quenching properties [44,45], and it is able to bind and inactivate redox-active iron ions [13], preventing hydroxyl radical production [34]. Neuromelanin has been shown to be a powerful chelator of neurotoxic metals and has both a high affinity and a low affinity chelation site for Fe^3+^, a high affinity and low affinity site [46]. In fact, NM appears to contain the largest reservoir of iron in catecholaminergic cells [29,30].

Ca^2+^ is another cation that coordinates catechols [33], but unlike Fe^3+^, does not initiate radical formation and did not drive melanogenesis. While it remains unclear if Ca^2+^ could disrupt/induce melanogenesis at abnormally high concentrations, the present results indicate that at physiologically relevant Ca^2+^ concentrations, melanogenesis is not dependent or enhanced by Ca^2+^. Interestingly, a key difference between DA cells in the ventral tegmental area (VTA) vs. SNc is that VTA DA cells have less cytosolic Ca^2+^ than do SNc DA cells due to calcium-based pacemaker channels [47], meaning that increased cytosolic Ca^2+^ is at least correlated with NM deposits. Furthermore, Lebedev et al. have given evidence for Ca^2+^ driving some forms of melanogenesis [48]. This appears to contrast with our data. However, our system largely isolated DA and Ca^2+^ from oxidative species and other sources for electron radicals. In the Mosharov study, cells were alive in in vivo conditions, which includes oxidative species [47], and in the Lebedev study, reactions were deliberately treated with potassium superoxide to initiate polymerization. Fe^3+^ can drive melanogenesis alone and Ca^2+^ can also drive melanogenesis, but only in the presence of an oxidizer. Thus, we propose that aggregation of DA around a coordinating cation and an oxidizer or radical are important components of spontaneous melanogenesis.

Like Fe^3+^, the pro-oxidant H_2_O_2_ increased the rate of melanogenesis. While high concentrations of H_2_O_2_ (~1 M) are able to degrade NM [34], the present concentration (500 μM) was below that threshold, potentially avoiding decomposition of melanin. While it appears that aggregation of DA molecules is important, it seems that with a high enough concentration of radicals, polymerization can proceed. Furthermore, this indicates that melanogenesis may function as an antioxidant in cells with free DA in the cytosol. Simply adding H_2_O_2_ is not enough to confirm that the presence of oxidative species can drive melanogenesis. H_2_O_2_ is particularly volatile and decomposes quickly and measuring accurate concentrations of H_2_O_2_ is difficult under reaction conditions.

Antioxidants are very stable in solution and easy to measure accurately. Ascorbate and GSH (natural antioxidants found in striatal tissue) both attenuated the rate of melanogenesis. Ascorbate is found throughout the brain in varying quantities dependent on anatomical location, with particularly high quantities in the striatum and in lower quantities in the midbrain tegmentum where DA neurons originate [36]. This suggests that in areas with high DA release and transport (i.e., striatum), there is also a high concentration of a melanogenesis blocker, potentially promoting DA recovery for re-release by preventing extracellular DA from turning into melanin in the synaptic cleft or in the cytosol of the terminal. This may explain why NM appears to form in greater amounts in cell bodies (tegmentum), but not in terminal areas (striatum). Glutathione is a small molecule that can function as a chemical antioxidant through its thiol group, spontaneously forming a disulfide bond with another GSH molecule in an oxidative environment, or as a substrate for the enzyme GSH peroxidase, which enzymatically joins two glutathione molecules through the same disulfide bond. Glutathione oxidation has been heavily implicated in DA cell health [49], indicating that DA cells are under a base load of oxidative stress and in need of managing concentrations of oxidative molecules. In fact, DAergic neurons require a higher baseline level of GSH (35 ± 3 nmol/mg in DAergic cells as compared to 8 ± 0.4 nmol/mg) for normal function [50].

We corroborated the effects of antioxidants on DA melanogenesis with FSCV in vitro and in the NAc of mouse brain slices ex vivo. Voltammetry enables high resolution spatiotemporal analysis of DA release with spatial resolution in the micrometer domain and temporal resolution in the sub-second time domain. Dopamine melanogenesis was observed in vitro with the same concentration used in the spectrophotometry studies. Voltammetry studies indicated that the oxidation current associated with infusion of 30 μM DA peaks quickly in a static system and then decays logarithmically over 1–2 h. If DA was not converted to melanin, it would equilibrate in a static system, which explains why the melanin current increases while DA current decreases over a 1–2 h period. Glutathione markedly reduced the formation of melanin and the progressive decrease in DA in vitro, suggesting that it is disrupting melanogenesis via its antioxidant properties. Second, in order to evaluate the physiological relevancy of these in vitro findings, we studied the effects of KCl on DA release and melanin formation in the NAc core region of mice using FSCV. The dose of KCl used to depolarize axon terminals in the NAc slice markedly enhanced DA to levels approaching that of synthetic DA in vitro. However, the variability was greater ex vivo, likely due to more variables associated with the biological tissue including the buffering and re-uptake of DA through the DA transporter (DAT). It is well known that drugs like cocaine or methylphenidate (Ritalin) that block the DAT or amphetamines that reverse the DAT markedly enhance DA release and that the DAT is operational in the slice preparation. Indeed, we have shown in multiple publications that cocaine and METH markedly enhance basal and evoked DA release [51,52,53,54,55,56,57,58], which is blocked by select antioxidants like ascorbate and GSH. The decrease in DA produced by KCl provides further evidence that the DAT was operational and that there was considerable DA re-uptake occurring in the face of melanogenesis. Indeed, although melanogenesis was evident, it was not as great as that produced by similar concentrations of DA in vitro. Notwithstanding differences in degree, GSH significantly reduced melanin formation produced by KCl-evoked DA release. However, it did not block the decrease in DA concentration over time as with 30 μM DA in vitro because of the ongoing uptake and reuptake of DA in the presence of tissue buffering and an operational DAT.

**Figure 7:**
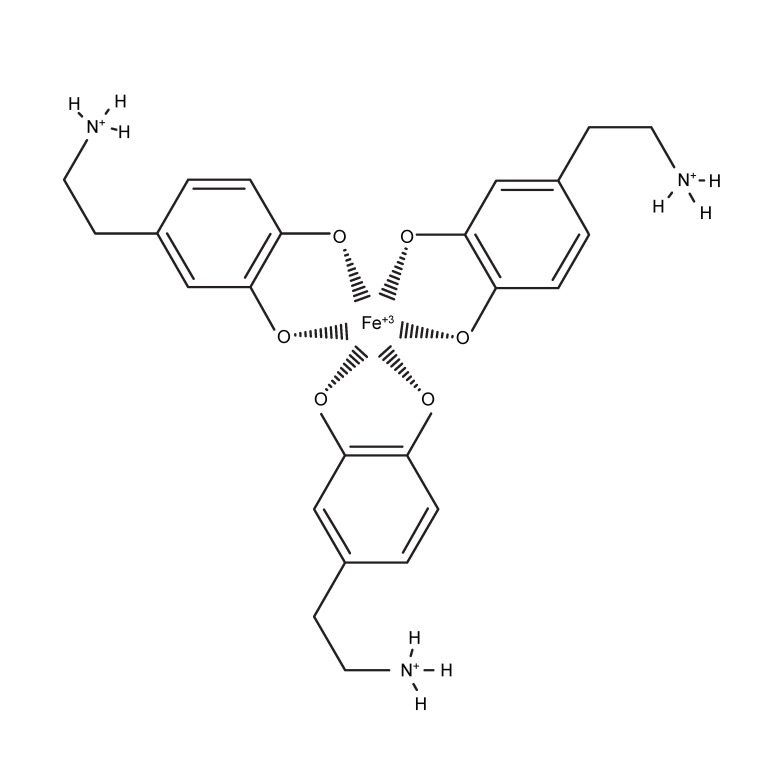
Proposed model for DA melanogenesis. To produce optimal formation of melanin, two factors are necessary: chelation and free-radical polymerization. Dopamine molecules chelate iron by the catechol group, positioning the DA molecules to allow for quick radical polymerization between the benzene rings, with block by radical scavenging antioxidants like ascorbate and GSH. The proposed model is that dopamine chelation of iron forms a dopamine aggregate around iron, bringing the benzene rings in close proximity to each other to allow for radical induced polymerization. While this may be the building block of neuromelanin, it should be noted that in vivo neuromelanin will include junk peptides, amino acids, and other biomolecules that will have been incorporated through proximity to radical polymerization.

We propose a model for DA melanogenesis (Figure 7). For the parameters investigated in this study, we speculate that DA molecules aggregate around a coordinating cation. Then, a radical can travel between the molecules, creating covalent bonds. This polymerization continues, creating chains of covalently-linked DA molecules (in varying states of oxidation), coordinated around Fe^3+^. Radical scavenger antioxidants would effectively block the polymerization catalyzed by Fe^3+^. While it is difficult to equate what happens in a test tube to the intricacies of the intracellular/extracellular environment, we propose that if there is a small volume of free DA in the cytosol of DA cell bodies, it will naturally aggregate around unsequestered Fe^3+^. Once aggregated, the spontaneous melanogenesis of DA to NM will occur when oxidative stress levels exceed a threshold, helping the cell to manage oxidative stress. Since melanins are insoluble, vacuoles will naturally pick up this polymer along with junk peptides, but will not be able to decompose it, resulting in vacuoles being filled with both a melanin and peptide component [59]. Over time, the number of these vacuoles increases in the cell, effectively pigmenting the entire cell body.

A growing body of literature identifies NM as a neuroprotective polymer [34], likely due to nascent antioxidant properties. For example, in models of PD, DA cells containing less NM are more susceptible to death than cells with increased NM pigmentation [11,12]. In this study, we demonstrate that melanin can spontaneous form from DA, especially in an environment that contains oxidative stress or Fe^3+^. We suggest that DA neurons use the unique chemistry of DA to manage oxidative stress. Oxidative stress from Fe^3+^ is minimized as once it is bound up in NM is redox-inactive [46,60,61] and, thus unable to catalyze the Fenton reaction. Oxidative stress formed as a byproduct of DA metabolism is prevented as excess DA is polymerized into NM instead of being degraded by monoamine oxidase. Finally, mature melanin is a functional free radical scavenger [34,44] and can directly decrease external sources of oxidative stress.

## 5. Conclusions

In this study, we have characterized the spontaneous polymerization of DA into a melanin chemically similar to NM. This chemical process is optimized in the presence of Fe^3+^ (but not Ca^2+^) or in the presence of pro-oxidative species such as H_2_O_2_ and is inhibited by antioxidants. Both Fe^3+^ and Ca^2+^ are known to aggregate DA molecules by chelating the catechol functional group, but only Fe^3+^ produced the polymerization. Through the Fenton reaction, Fe^3+^ drives the formation of oxidative species, indicating that oxidative species are necessary for DA melanogenesis and that DA aggregation while chelated around a polyvalent cation is insufficient to trigger spontaneous melanogenesis. Finally, when aggregated and in the presence of an oxidative species, DA polymerizes into a melanin in a rapid, thermodynamically stable reaction. Melanization occurs in ex vivo conditions which is blocked by antioxidants. Although the effects of melanization in vivo are still not fully understood, the present results indicate that melanization requires pro-oxidants. Thus, melanization may more relevant under conditions where iron has breached the blood brain barrier, such as ischemia, or increases in local H_2_O_2_, which has been implicated as a retrograde signaling molecule but is also present in intracellular compartments.

## Supplementary Materials

The following are available online at https://www.mdpi.com/2076-3921/9/12/1285/s1, Figure S1: H-NMR spectra showing that the dominant species at the (A) beginning of the reaction is dopamine, and (B) still dopamine at the end of the reaction. (C) Overlay of the spectra in (A) and (B) demonstrating that the peaks match perfectly, Figure S2: Calcium does not contribute to dopamine (DA) melanization, Figure S3: The antioxidant GSH reduces dopamine melanogenesis in a concentration dependent manner.

## Abbreviations

DA	Dopamine
DES	Desferoxamine
DOPAL	3,4-dihydroxyphenylacetaldehyde
ECF	Extracellular Fluid Buffer
GSH	Glutathione
H-NMR	Proton Nuclear Magnetic Resonance
ICF	Intracellular Fluid Buffer
NM	Neuromelanin
PD	Parkinson’s Disease
SNc	Substantia Nigra Pars Compacta
VTA	Ventral Tegmental Area
