# Supplementary material for: Spontaneous Formation of Melanin from Dopamine in the Presence of Iron
Source: Antioxidants (Basel). 2020 Dec 16;9(12):1285. doi: 10.3390/antiox9121285 (PMC7766172; doi:10.3390/antiox9121285)
Supplement: Supplementary file 1 [file antioxidants-09-01285-s001.pdf]

Supplemental

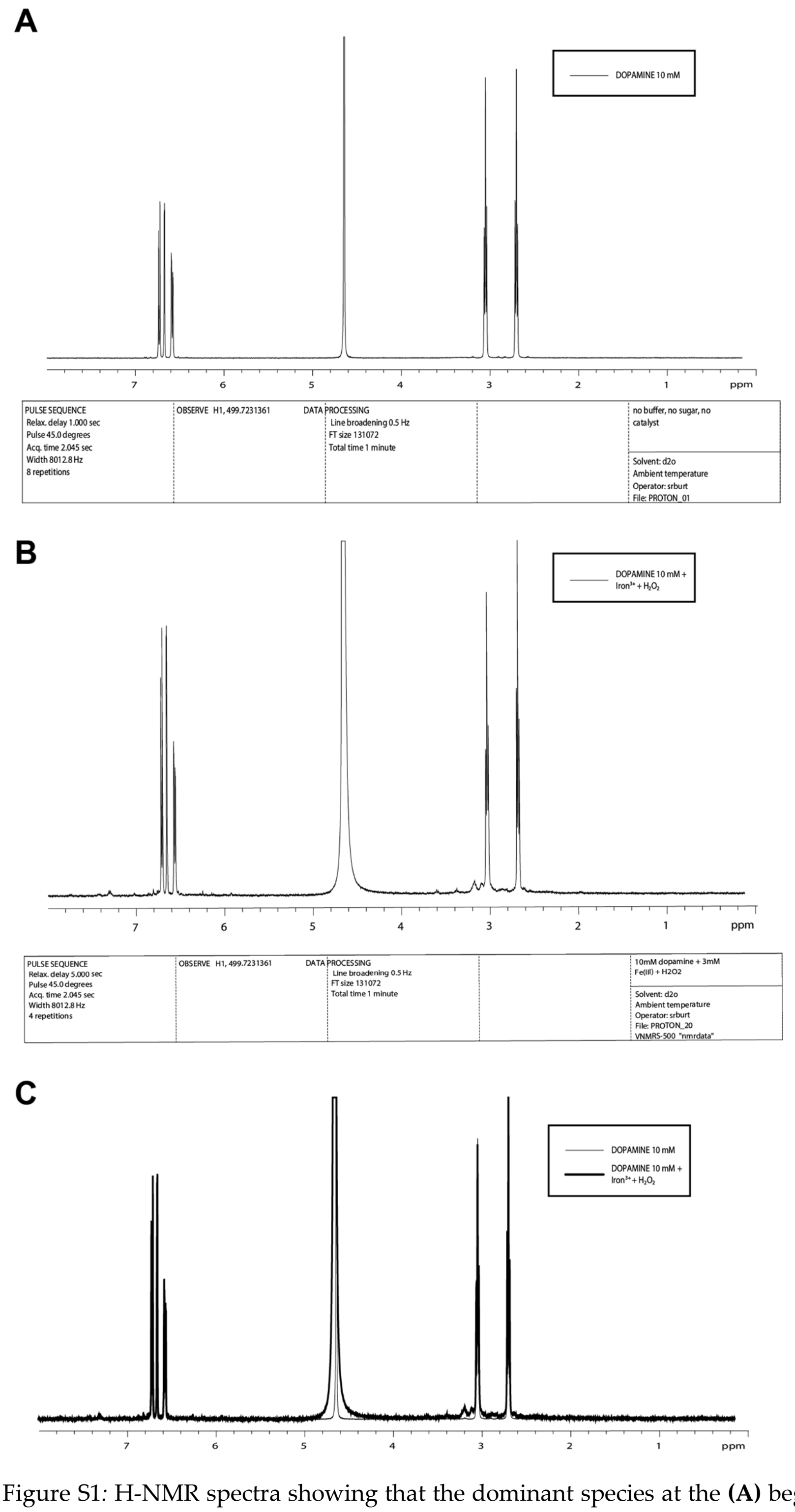


Figure S1: H-NMR spectra showing that the dominant species at the **(A)** beginning of the reaction is dopamine, and **(B)** still dopamine at the end of the reaction. **(C)** Overlay of the spectra in **(A)** and **(B)** demonstrating that the peaks match perfectly.

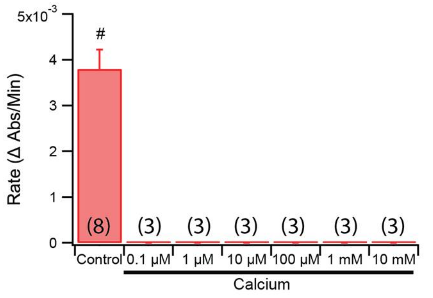


Figure S2: Calcium does not contribute to dopamine (DA) melanization. Control conditions are in the presence of trace elemental $Fe^{3+}$ and indicate melanization with DA (30 µM). For the increasing calcium conditions, $Fe^{3+}$ was chelated using transferrin prior to addition of increasing $Ca^{2+}$ concentrations. No melanogenesis was observed for calcium experiments. Significance marker # denotes $p< 0.0001$ significant differences for control vs all $Ca^{2+}$ conditions as determined by a Tukey's HSD pairwise comparison. Error bars are mean + SEM. The n value for experiments is listed in parenthesis.

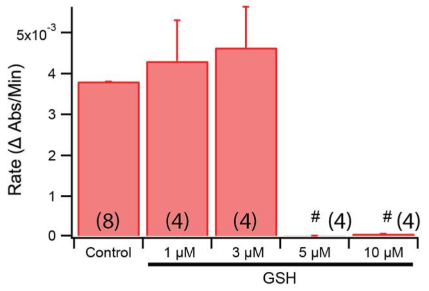


Figure S3: The antioxidant GSH reduces dopamine melanogenesis in a concentration dependent manner. Rate of melanin formation was comparable under control conditions, and with lower concentrations of GSH (1-3 µM). However, higher GSH concentrations similar to those used in Fig 4 (5-10 µM) blocked melanin formation. The binary nature of this effect indicates that the effect concentrations required are higher than 3 µM. Significance marker # denotes $p< 0.0001$ significant differences for control, 1 and 3 µM vs 5 and 10 µM. GSH conditions as determined by a Tukey's HSD pairwise comparison. Error bars are mean + SEM. The n value for experiments is listed in parenthesis.
